# Safety and Efficacy of 2D Brachytherapy vs. 3D Image-Guided Adaptive Brachytherapy for Locally Advanced Cervical Cancer—A Single Institution Retrospective Study

**DOI:** 10.3390/curroncol30050375

**Published:** 2023-05-13

**Authors:** Mame Daro Faye, Mariana Petruccelli Araujo, Michel D. Wissing, Khalid Alrabiah, Lucy Gilbert, Xing Zeng, Luis Souhami, Joanne Alfieri

**Affiliations:** 1 Division of Radiation Oncology, McGill University Health Centre, Montreal, QC H4A 3J1, Canada; mame.faye@mail.mcgill.ca (M.D.F.);; 2 Division of Cancer Epidemiology, Oncology, McGill University, Montreal, QC H4A 3T2, Canada; 3 Department of Gynecology-Oncology, McGill University Health Centre, Montreal, QC H4A 3J1, Canada

**Keywords:** cervical cancer, radiation oncology, radiotherapy, brachytherapy, 3D-IGABT

## Abstract

Background: The treatment paradigm for locally advanced cervical cancer (LACC) has shifted from two-dimensional-brachytherapy (2D-BT) to three-dimensional-image-guided adaptive BT (3D-IGABT). In this retrospective study, we report our experience with the change from 2D-BT to 3D-IGABT. Methods: We reviewed 146 LACC patients (98 3D-IGABT and 48 2D-BT) who received chemoradiation between 2004 and 2019. The multivariable odds ratio (OR) for treatment-related toxicities and hazard ratios (HR) for locoregional control (LRC), distant control (DC), failure-free survival (FFS), cancer-specific survival (CSS) and overall survival (OS) are reported. Results: The median follow-up was 50.3 months. There was a significant decrease in overall late toxicities in the 3D-IGABT group compared to the 2D-BT group (OR 0.22[0.10–0.52]), late gastrointestinal (OR 0.31[0.10–0.93]), genitourinary (OR 0.31[0.09–1.01]) and vaginal toxicities (0% vs. 29.6%). Grade ≥ 3 toxicity was low in both groups (2D-BT: 8.2% acute, 13.3% late vs. 3D-IGABT: 6.3% acute, 4.4% late, NS). The five-year LRC, DC, FFS, CSS and OS for 3D-IGABT were 92.0%, 63.4%, 61.7%, 75.4% and 73.6%, compared to 87.3%, 71.8%, 63.7%, 76.3% and 70.8% for 2D-BT (NS). Conclusions: 3D-IGABT for the treatment of LACC is associated with a decrease in overall late gastrointestinal, genitourinary and vaginal toxicities. The disease control or survival outcomes were comparable to contemporary 3D-IGABT studies.

## 1. Introduction

The standard treatment of locally advanced cervical cancer (LACC) consists of concurrent chemoradiotherapy (CCRT) and external beam radiotherapy (EBRT), followed by brachytherapy (BT) [1]. Brachytherapy (BT) is a crucial component of the management of LACC and, when combined with chemoradiotherapy, has been shown to improve local control and overall survival [2,3]. With the advent of new treatment modalities and imaging technologies in radiotherapy, treatment paradigms have shifted from the use of 2D brachytherapy (2D-BT) with orthogonal X-rays to that of 3D image-guided adaptive brachytherapy (3D-IGABT) using computed tomography (CT) or magnetic resonance imaging (MRI). The goal of 3D-IGABT is to deliver doses more precisely to clinical target volumes, as well as organs at risk (OARs) [4]. Indeed, 2D-BT prescribes a pear-shaped dose to a point A regardless of tumour size, anatomy or doses to OARs, whereas 3D-IGABT takes into account variations in tumour size and position over the treatment course for conformal treatment of a high-risk clinical target volume (HR-CTV) while simultaneously sparing OARs [5,6].

Overall, 3D-IGABT has now become the new standard for BT boost in the treatment of LACC as several studies have reported improved treatment outcomes and decreased toxicities from this approach [7,8,9]. At our institution, cone beam CT (CBCT) was introduced in 2008 as an alternative to orthogonal X-rays to plan and deliver 2D-BT, with a dose prescription to point A and reporting of International Commission on Radiation Units and Measurements (ICRU) bladder and rectum doses [10]. Comparing these ICRU dose estimates to dose-volume histograms (DVHs) derived from CBCT, Al-Halabi et al. [11] showed that median ICRU bladder and rectal doses were significantly lower than D2cc bladder and rectal doses for the same patients and underestimate the true maximal dose to OARs. Institutional practices later changed to the use of helical CT-guided BT in 2012 and MRI-guided BT routinely implemented in 2015. Thus, in this retrospective study, we aim to report the safety and efficacy outcomes of cervical cancer patients treated with 3D-IGABT vs. ICRU-based 2D-BT at the McGill University Health Centre (MUHC) between 2004 and 2019. We hypothesize that LACC patients treated with 3D-IGABT have less treatment-related toxicities and possibly improved locoregional control (LRC), failure-free survival (FFS), cancer-specific survival (CSS) and overall survival (OS) than those treated with 2D-BT.

## 2. Materials and Methods

### 2.1. Study Design and Patient Population

This is a retrospective observational study of all patients with histologically confirmed International Federation of Gynecology and Obstetrics (FIGO) stage IB—IVB (oligometastatic) cervix cancer who received curative intent treatment by definitive EBRT ± concurrent chemotherapy followed by BT at the MUHC from February 2004 to August 2019. A total of 242 patients were treated; 94 were excluded due to charts not being electronically available and two patients were excluded for having a contraindication for BT, leaving a total of 146 patients for analysis. This included 48 patients treated with 3D-IGABT and 98 with 2D-BT (Table 1). Data on patient demographics, clinicopathologic characteristics, treatments, treatment-related side effects, and clinical outcomes were retrospectively collected from medical records until December 2019. The study was approved by the institutional ethics committee (REB approval # 2022-7783). 

### 2.2. Treatment Delivery and Follow-Up

Patients were clinically staged according to the FIGO staging systems 2000 or 2009 [12,13]. Tumours were assessed based on physical examination, imaging and staging carried out by CT of the chest, abdomen and pelvis, MRI of the pelvis and/or PET/CT scan. Surgical lymph node staging was not an exclusion criterion. Treatment consisted of EBRT with a median dose of 45 Gy in 25 fractions (range: 45–55 Gy) over 5 weeks delivered to the pelvis and para-aortic region (if para-aortic positive node) ± concurrent chemotherapy (most commonly weekly cisplatin 40 mg/m^2^), followed by a high dose rate BT. A nodal boost was given to metabolically active and/or radiologically abnormal pelvic and para-aortic positive lymph nodes. Delivery of EBRT was either by 3D conformal four-field technique for older cohorts or intensity modulated radiotherapy (IMRT) as of the year 2007. BT, performed under spinal anaesthesia, was mostly delivered in 3 weekly fractions of 8 Gy. Mean overall treatment time was 45 ± 7.69 days (range 24–73 days). Overall, 132/146 (90.4%) patients were treated with tandem and ovoids. In 14/146 patients (9.6%), a cylinder, ring or needles were used and specifically 4/146 patients (2.7%) had interstitial brachytherapy. CBCT, CT and/or MRI for treatment planning were performed at every fraction. A CBCT-based ICRU/2D-BT plan was performed for 99/100 patients (99%) treated between February 2004 and July 2013. Patients treated between August 2013 and February 2016 (19/20 patients, 95%) received CT-guided volume-based planning, and all those treated between February 2016 and August 2019 (26 patients) received MRI-guided planning as per the Groupe Européen de Curiethérapie and the European Society for Radiotherapy & Oncology (GEC-ESTRO) recommendations [14]. The 3D-IGABT was performed as per the Embrace II protocol [15,16,17].

Patients were followed weekly during radiotherapy and potential side effects were addressed and prospectively recorded according to the Common Terminology Criteria for Adverse Events (CTCAE) version 4.03 [18]. Patients were then followed with history and physical examinations q3 months for the first 2 years, q6 months for the next 3 years, and q12 months thereafter. FDG-PET was performed 3 months after end of treatment and then as indicated. CT restaging was carried out yearly as part of routine surveillance and MRIs of the pelvis were performed as indicated by symptoms. Survival, tumour recurrence, and complications were measured from the start date of EBRT to the date of the event, death, or last follow-up. 

### 2.3. Study Endpoints and Statistical Analysis

The primary endpoint of the study was toxicity profile, defined as the incidence of any acute or late treatment-related toxicities. These included any genito-urinary (GU), gastro-intestinal (GI), haematological, vaginal (vaginal atrophy, dyspareunia, post-coital bleeding), fatigue, pain and dermatological/neurological toxicities. Acute toxicities were defined as treatment-related toxicities that occurred during or within 6 months of the end of treatment and late toxicities as those occurring 6 months or more from the end of treatment. Secondary endpoints included locoregional control (LRC), distant control (DC), failure-free survival (FFS), cancer-specific survival (CSS) and overall survival (OS) at 3 and 5 years. LRC was defined as the absence of disease within the pelvis as assessed clinically and/or by biopsy or imaging. DC was defined as the absence of extra-pelvic nodal and/or organ metastases, including para-aortic/retroperitoneal lymph nodes. FFS was defined as any absence of locoregional or distant progression/recurrence. Cause of death was documented to determine CSS and OS rates. 

Univariable and multivariable ordered-logistic regression models were used to calculate odds ratios (OR) and their respective 95% confidence intervals for acute and late toxicities in the 3D-IGABT compared to the 2D-BT arms, taking into account the incidence and grade of toxicities [19]. Survival outcomes were computed using the Kaplan–Meier method and a *p*-value < 0.05 was regarded as statistically significant [20]. Cox proportional hazard regression model (univariable and multivariable) was used to derive hazard ratio (HR) [19], adjusted for choice of BT, age, histology, stage, lymph node involvement, EBRT and BT doses. 

## 3. Results

The median follow-up time was 50.3 months (95% CI 35.6–57.6) for the whole cohort measured by the reverse Kaplan–Meier method [21], 69.1 months (95% CI 56.2–79.3) for the 2D-BT group and 25.3 months (95% CI 15.1–34.4) for the 3D-IGABT group. 

### 3.1. Patient Characteristics

The patient and treatment characteristics of the 3D-IGABTand 2D-BT groups are presented in Table 1. There were no statistically significant differences for any of the patient characteristics.

### 3.2. Acute and Late Treatment-Related Toxicities 

Differences in treatment-related toxicities were evaluated in the 3D-IGABT and 2D-BT groups by ordered logistic regression, taking into account both the incidence and grade of toxicities. Two 3D-IGABT patients were excluded from the late toxicity analysis as they did not reach a long enough follow-up time for this. Table 2 summarizes these findings. There was no difference in cumulative acute toxicity events, or in any GI or GU acute toxicities experienced in each group. However, there was a significant decrease in acute vaginal toxicities in the 3D-IGABT group (OR 0.08 (95% CI 0.01–0.60)) compared to the 2D-group, with only 1/48 patient (2.1%) in the 3D-IGABT reporting dyspareunia and post-coital bleed, compared to 21/98 (21.4%) in the 2D-BT group with similar symptoms. This difference was maintained on multivariable analysis (MVA) when adjusted for age, stage, dose of EBRT or BT. There was a significant increase in acute haematological toxicities in the 3D-IGABT group (OR 2.50 (95% CI 1.05–5.98)), although this difference was not maintained on MVA (OR 2.67 (95% CI 0.97–7.39)).

There was a statistically significant decrease in the total number and grade of late treatment-related toxicities in the 3D-IGABT compared to the 2D-BT group (OR 0.25 (95% CI 0.12–0.54)) and this difference was maintained on MVA (OR 0.22 (95% CI 0.10–0.52)). In particular, there was a significant decrease in the incidence and grade of overall late GI toxicities in the 3D-IGABT group (OR 0.31 (95% CI 0.10–0.93) on MVA) with 6/46 (13%) patients in the 3D-IGABT group experiencing late GI toxicities compared to 26/98 (26.5%) patients in the 2D-BT group. There was also a decrease in overall late GU toxicities in the 3D-IGABT group on MVA (OR 0.31 (95% CI 0.09–1.01)). No patients in the 3D-IGABT experienced late vaginal toxicities compared to 29/98 (29.6%) patients in the 2D-BT group. The rate of acute and late grade ≥ 3 toxicities was 6.3% and 4.4%, respectively, in the 3D-IGABT group, compared to 8.2% and 13.3% in the 2D-BT group. Grade ≥ 3 late GI toxicities were recorded in 2/46 (4.4%, grade 3 colitis) 3D-IGABT patients compared to 9/98 (9.2%) 2D-BT patients, including four cases of large bowel obstruction one of which required urgent surgery (grade 4), grade 3 bowel fistulas (2), grade 3 colitis (1), grade 3 proctitis (1) and grade 3 abdominal pain without colitis (1). Grade ≥ 3 late GU toxicities were recorded in 0/46 (0%) 3D-IGABT patients and 3/98 (3%) 2D-BT patients with all three cases being grade 3 vesicovaginal fistulas. There was no statistically significant difference in grade ≥ 3 acute and late toxicities between the two groups, but the number of such events was small (Table 3). Importantly, there was no grade 5 toxicity. 

### 3.3. Locoregional and Distant Disease Control 

**Figure 1:**
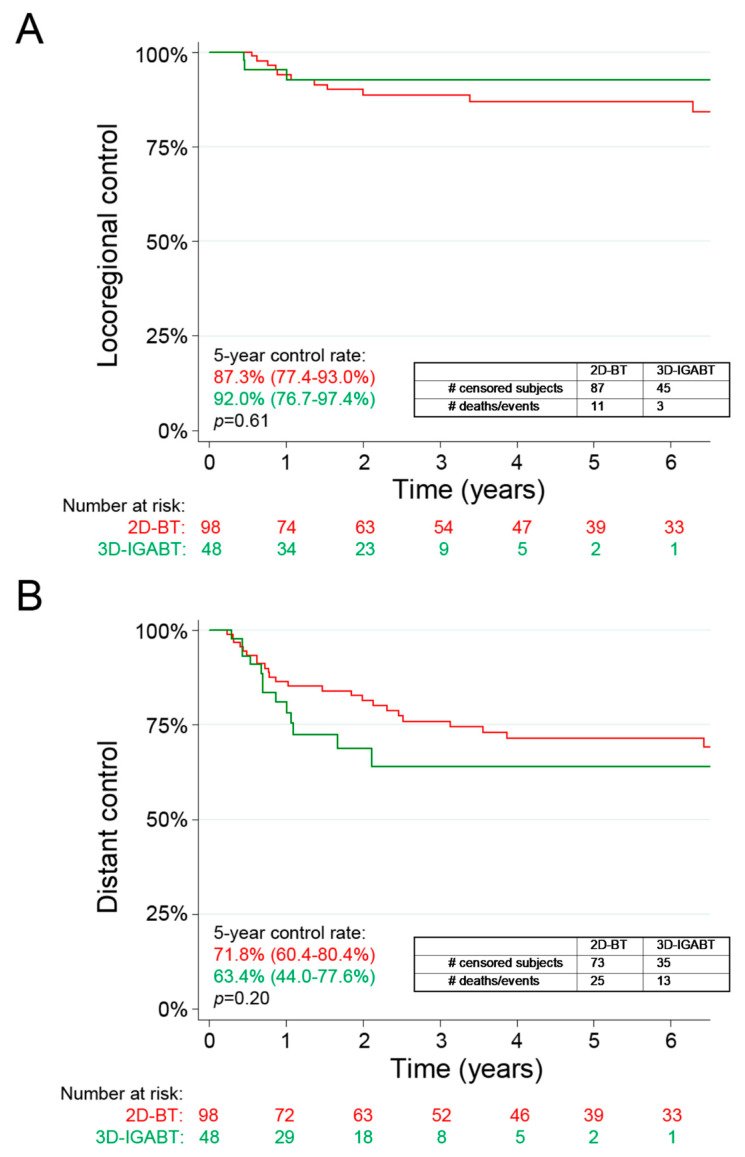
Locoregional (**A**) and distant (**B**) control rates in patients treated with 3D-IGABT vs. 2D-BT. 2D-BT = 2D brachytherapy, 3D-IGABT = 3D Image-guided adaptive brachytherapy. Kaplan–Meier estimates for locoregional (**A**) and distant failure events (**B**) over time. Numbers at risk are displayed. *p*-values were calculated using log-rank tests.

The 5-year LRC rate was 92.0% in the 3D-IGABT group, compared to 87.3% in the 2D-BT group (*p* = 0.61, Figure 1A). HR for LRC was 0.81 (95% CI 0.21–3.13) for the 3D-IGABT compared to the 2D-BT on MVA (Table 4), when adjusted for age, histology, clinical stage, location of positive lymph nodes and EBRT/BT doses. The 3-year LRC rates were similar (Table S1). Surprisingly, DC was worse in the 3D-IGABT group with 5-year DC of 63.4% compared to 71.8% in the 2D-BT group, although not statistically significant (*p* = 0.20, Figure 1B). HR for DC was 1.94 (95% CI 0.85–4.40) for the 3D-IGABT compared to the 2D-BT on MVA. Two patients in each group had synchronous locoregional and distant failures. 

### 3.4. Survival Outcomes 

**Figure 2:**
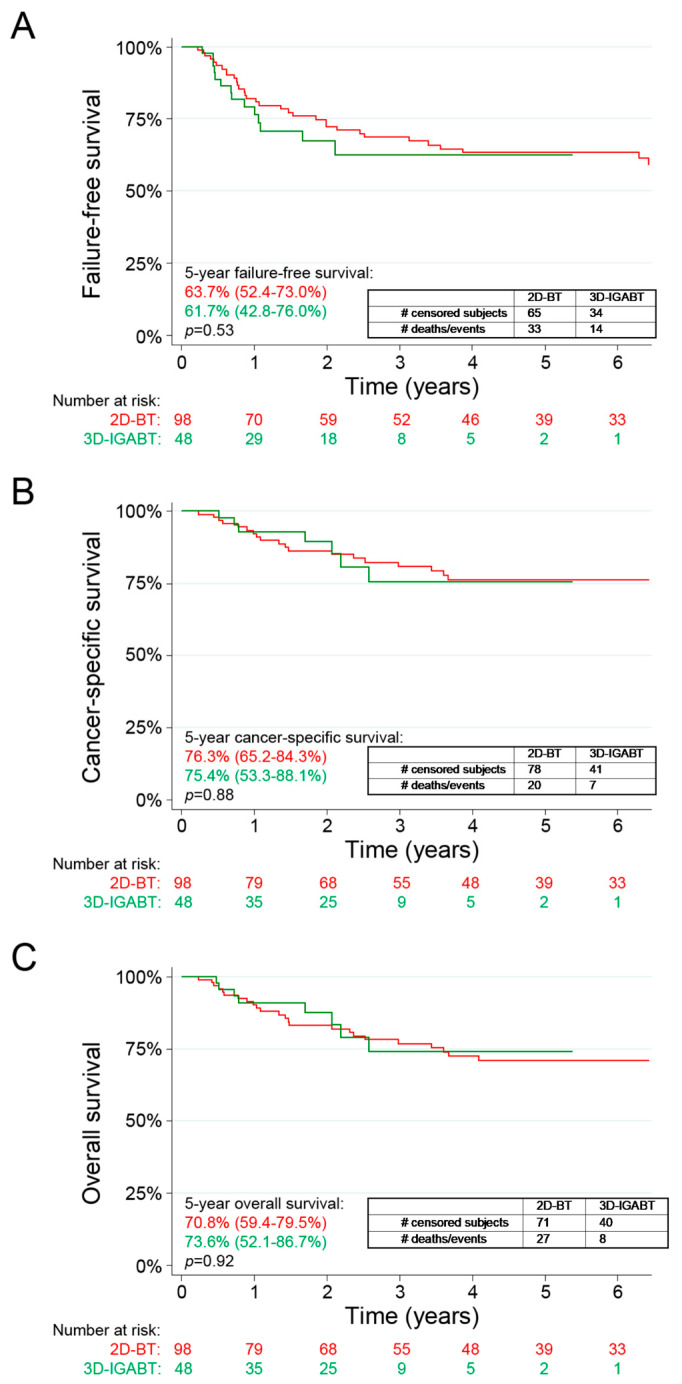
Failure-free survival (**A**), cancer-specific survival (**B**) and overall survival (**C**) in patients treated with 3D-IGABT vs. 2D-BT. 2D-BT = 2D brachytherapy, 3D-IGABT = 3D Image-guided adaptive brachytherapy. Kaplan–Meier estimates for failure-free survival (**A**), cancer-specific survival (**B**) and overall survival (**C**). Numbers at risk are displayed. *p*-values were calculated using log-rank tests.

There were no statistically significant differences in FFS, CSS or OS between the two treatment groups. The 5-year FFS rate was 61.7% for the 3D-IGABT, compared to 63.7% in the 2D-BT group (*p* = 0.53, Figure 2A). At the time of the last follow-up, there were a total of 35/146 (24%) deaths, including 8/48 (16.7%) patients in the 3D-IGABT group and 27/98 (27.6%) patients in the 2D-BT group. Six patients were diagnosed with another primary cancer during the follow-up period, three of which were cured from their cervical cancer but passed away from the second primary cancer. Importantly, none of these cancers were in the treatment field. The 5-year CSS was 75.4% in the 3D-IGABT group compared to 76.3% in the 2D-BT (*p* = 0.88, Figure 2B). The 5-year OS rates were 73.6% in the 3D-IGABT compared to 70.8% in the 2D-BT group (*p* = 0.92, Figure 2C). 

### 3.5. Factors Associated with Worse Disease Control and Survival Outcomes 

Table 4 shows the factors associated with worse disease control and survival outcomes in this population on uni- and multivariable Cox regression, adjusting for choice of RT, age, histology, clinical stage, location of positive lymph nodes, and EBRT/BT dose. A higher clinical stage was significantly predicted for worse DC, FFS, CSS and OSS both on uni- and multivariable analysis (Table 4, Figure S1). Having positive lymph nodes also independently significantly predicted an increased risk of DC and survival. In particular, having positive pelvic lymph nodes was associated with a four-fold increased risk of distant failure and a nine-fold increase in death specifically from the disease. Having positive para-aortic lymph nodes was associated with seven-fold more distant failures, twelve-fold more disease-specific deaths and a five-fold greater overall risk of death on MVA. Having had cisplatin concurrently with EBRT was associated with a significantly improved FFS, as well as CSS and OS survival benefits (Table 4). Interestingly, increased age at diagnosis was associated with improved local control (HR = 0.66 (95% CI 0.46–0.94) on MVA, Table 4).

## 4. Discussion

In this study, we are reporting on the safety and efficacy of 3D-IGABT compared to 2D-BT in a cohort of LACC patients treated with curative intent at the MUHC between 2004 and 2019. We found a significant decrease in the total number and grade of late treatment-related toxicities in favour of the 3D-IGABT and this was particularly evident for late GI, GU and vaginal toxicities. These findings are in line with previous reports of the superior safety of 3D-IGABT compared to 2D-BT in the treatment of cervical cancer. Indeed, the first prospective, non-randomized trial comparing 2D- vs. 3D-BT in LACC, the French STIC trial, reported a 50% reduction in grade 3 and 4 morbidity in favour of 3D-BT [22]. In a meta-analysis on the outcomes of 3D- vs. 2D- intracavitary BT, Kim et al. [9] confirmed the superior safety profile of 3D-IGABT with a reported pooled HR for grade ≥ 3 toxicities of 0.54 (95% CI 0.37–0.77) [9]. We also found a clinical difference in grade ≥ 3 late toxicities in the 3D-IGABT vs. 2D-BT groups, although this did not reach significance (4.4% and 13.3%, respectively, OR 0.30[0.06–1.38], Table 3). This is likely due to the relatively low incidence of grade 3 and 4 toxicities recorded in our cohort. In comparison, Lin et al. reported late grade ≥ 3 toxicities in 11% of patients in their IMRT/3D-IGABT cohort compared to 18% in the 2D EBRT/BT cohort [8]. In the RetroEMBRACE study of 3D-IGABT, 5-year grade ≥ 3 toxicity was 5%, 7% and 5%, respectively, for the bladder, GI tract and vagina [7]. The recently published EMBRACE I prospective study on MRI-based IGABT showed an actuarial 5-year grade ≥ 3 toxicity of 6.8% for GU events, 8.5% for GI events, 5.7% for vaginal events [23]. In comparison, we found late grade 3–4 toxicities of 4.4%, 0%, 0% for the bladder, GI tract and vagina, respectively, in our 3D-IGABT cohort (Table 3). It is, however, important to note that the cut-off for late toxicity was 3 months for EMBRACE I whereas in our study, we recorded late toxicities starting at 6 months, which could have potentially underestimated the rate of late toxicities although minimally. 

The LRC rates in both treatment groups were comparable to previous reports, although we did not find a statistically significant difference of LRC with the use of 3D-IGABT. The 5-year LRC rate was 92.0% in the 3D-IGABT group, compared to 87.3% in the 2D-BT group (*p* = 0.61, Figure 1A). In comparison, EMBRACE I reported an actuarial 5-year LC of 92.0% at a median follow-up of 51 months and the RetroEMBRACE study reported 5-year LC rates of 89% using 3D-IGABT [7]. Using the modern techniques of PET-CT simulation and MRI-guided BT, Lin et al. [8] also did not find any significant difference in LRC when comparing IMRT/3D-IGABT to 2D-EBRT/BT, with 5-year LRC rates of 81% and 78%, respectively (*p* = 0.5). We hypothesize that such variability in the added benefit of 3D-IGABT is multifactorial and will depend on the patient population and stage distribution, tumour size, techniques used (CT vs. MRI guidance, interstitial needles) and operator experience. The advantage of 3D-IGABT over 2D-BT on survival outcomes is equivocal in studies making a direct comparison between the modalities. Indeed, some studies have shown a significant improvement in PFS/FFS [8] and OS [8,24], whereas in other studies the improvement was not significant [22,25,26,27]. A recent meta-analysis found a significant improvement in pooled LRRFS (HR = 0.61 (95% CI 0.40–0.93)) and PFS (HR = 0.75 (95% CI 0.59–0.96)) favouring 3D-IGABT, but not in OS (HR = 0.65 (95% CI 0.40–1.06)) [9]. We did not find any statistically significant difference in FFS, CSS and OS between the two treatment modalities, but the rates were comparable to those reported in the literature in terms of efficacy. The 5-year FFS and OS were 61.7% and 73.6%, respectively, in our 3D-IGABT cohort compared to a 5-year disease-free survival of 68% and 5-year OS of 74% in EMBRACE I. Thus, our institutional results using 3D-IGABT, although retrospective, compare favourably to those reported in the EMBRACE-I trial, both in terms on treatment efficacy and safety. 

There are some limitations to this retrospective study including the risks for selection, recall, underreporting and confounding biases. Moreover, because we compared the outcomes of two techniques that were sequentially established, the 3D-IGABT group inevitably had a shorter follow-up time than the 2D-BT group. It is also difficult to differentiate the added benefit that IMRT planning had on the improved toxicity profile of 3D-IGABT because patients in the 3D-IGABT group all received IMRT-based EBRT, whereas only ~30% in the 2D-BT did. Finally, our cohort sample size may have been too small to capture statistically significant differences in the local control and survival outcomes between the two groups. Nevertheless, this retrospective study allowed us to assess how our practice compared to the literature in terms of safety and survival outcomes of 3D-IGABT for LACC and add to the body of literature on 3D-IGABT.

## 5. Conclusions

Overall, 3D-IGABT for the treatment of cervical cancer was associated with a decrease in the rate and grade of late toxicities, specifically late gastrointestinal, genitourinary and vaginal toxicities, thus again confirming 3D-IGABT as a safer treatment approach for cervical cancer. The survival outcomes in our cohort compared favourably to those of larger prospective studies using 3D-IGABT, notably the recently published EMBRACE-I trial.

## Figures and Tables

**Table 1:** Baseline characteristics.

	2D-BT (*n* = 98)	3D-IGABT (*n* = 48)	*p*-Value
Age at diagnosis (Mean (SD))	52 (13)	54.7 (14.6)	0.20
Histology at biopsy			0.45
− Squamous cell carcinoma (SCC)	87 (88.8%)	40 (83.3%)	
− Adenocarcinoma	7 (7.1%)	5 (10.4%)	
− Clear cell	0 (0.0%)	1 (2.1%)	
− Other	3 (3.1%)	2 (4.2%)	
− Missing	1 (1.0%)	0 (0.0%)	
Stage			0.88
− 1	13 (14.3%)	8 (16.7%)	
1B1	10 (10.2%)	6 (12.5%)	
1B2	3 (3.1%)	2 (4.2%)	
− 2	50 (51.0%)	26 (54.2%)	
2A1	3 (3.1%)	2 (4.2%)	
2A2	5 (5.1%)	1 (2.1%)	
2B	43 (42.9%)	23 (47.9%)	
− 3	21 (21.4%)	8 (16.7%)	
3A	2 (2.0%)	0 (0.0%)	
3B	19 (19.4%)	8 (16.7%)	
− 4	14 (14.3%)	6 (12.5%)	
4A	6 (6.1%)	2 (4.2%)	
4B	8 (8.2%)	4 (8.3%)	
Lymph node status			
− Positive lymph nodes	48 (49.0%)	32 (66.7%)	0.13
Pelvic	29 (29.6%)	21 (43.8%)	0.28
Para-aortic/Retroperitoneal	19 (19.4%)	11 (22.9%)	
− Negative lymph nodes	42 (42.9%)	16 (33.3%)	
− Unknown	8 (8.2%)	0 (0%)	
PET staging			0.20
− Yes	77 (78.6%)	43 (89.6%)	
− No	11 (11.2%)	5 (10.4%)	
− Unknown	10 (10.2%)	0 (0%)	
Treatment duration	44 (42-50)	46 (42-51)	0.33
− >8 weeks	8 (8.2%)	8 (16.7%)	0.12
EBRT dose			0.09
− ≤50.4 Gy	92 (93.9%)	41 (85.4%)	
− >50.4 Gy	6 (6.1%)	7 (14.6%)	
Brachytherapy dose			0.62
− ≥24 Gy	85 (86.7%)	43 (89.6%)	
− <24 Gy	13 (13.3%)	5 (10.4%)	
Concurrent chemotherapy	45 (93.8%)	88 (89.8%)	0.43
− Unknown chemotherapy regimen	2 (2.0%)	1 (2.1%)	
− Cisplatin	85 (86.7%)	40 (83.3%)	0.56
− Gemcitabine	2 (2.0%)	4 (8.3%)	0.07

2D-BT = 2D brachytherapy, 3D-IGABT = 3D Image-guided adaptive brachytherapy, EBRT = external beam radiotherapy. Continuous variables are displayed as the median (interquartile range), and categorical variables as the patient number (percentage).

**Table 2:** Comparison of treatment-related toxicities in patients treated with 3D-IGABT vs. 2D-BT.

			Odds Ratio	
	2D-BT (*n* = 98)	3D-IGABT (*n* = 48)	Univariable	Multivariable ^1^
Early toxicities				
Any	62 (63.3%)	33 (68.8%)	1.19 (0.63–2.23)	1.25 (0.62–2.52)
GI	31 (31.6%)	20 (41.7%)	1.56 (0.78–3.14)	2.01 (0.92–4.41)
GU	21 (21.4%)	9 (18.8%)	0.83 (0.35–1.98)	0.87 (0.35–2.18)
Haematological	12 (12.2%)	13 (27.1%)	**2.50** **(1.05–5.98)**	2.67 (0.97–7.39)
Fatigue/pain	30 (30.6%)	11 (22.9%)	0.62 (0.28–1.37)	0.56 (0.23–1.34)
Dermatological/neurological	7 (7.1%)	5 (10.4%)	1.52 (0.46–5.06)	1.45 (0.41–5.11)
Vaginal	21 (21.4%)	1 (2.1%)	**0.08** **(0.01–0.60)**	**0.07** **(0.01–0.62)**
Late toxicities				
Any	56 (57.1%)	12 (26.1%)	**0.25** **(0.12–0.54)**	**0.22** **(0.10–0.52)**
GI	26 (26.5%)	6 (13.0%)	0.41 (0.16–1.07)	**0.31** **(0.10–0.93)**
GU	22 (22.5%)	4 (8.7%)	**0.32** **(0.10–0.99)**	0.31 (0.09–1.01)
Haematological	4 (4.1%)	1 (2.2%)	0.52 (0.06–4.74)	0.81 (0.08–8.39)
Fatigue/pain	13 (13.3%)	5 (10.9%)	0.77 (0.26–2.31)	1.10 (0.33–3.66)
Dermatological/neurological	2 (2.0%)	1 (2.2%)	1.07 (0.09–12.07)	2.25 (0.07–68.33)
Vaginal	29 (29.6%)	0 (0.0%)	**0**	N/A

2D-BT = 2D brachytherapy, 3D-IGABT = 3D Image-guided adaptive brachytherapy. ^1^ Adjusted for: age (continuous), histology (categorical), stage (1/2/3/4, continuous), lymph node involvement (binary), EBRT dose (categorical), and brachytherapy dose (categorical). Uni- and multivariable ordered logistic regression models were used to calculate odds ratios and their respective 95% confidence intervals, taking into account both the incidence and grade of toxicities. An odds ratio above 1 means more toxicity in the 3D-IGABT group, and below 1 less toxicity. Significant values (*p* < 0.05) are marked in bold.

**Table 3:** Comparison of grade ≥ 3 treatment-related toxicities in patients treated with 3D-IGABT vs. 2D-BT.

			Odds Ratio	
	2D-BT (*n* = 98)	3D-IGABT (*n* = 48)	Univariable	Multivariable ^1^
Early toxicities				
Any	8 (8.2%)	3 (6.3%)	0.75 (0.19–2.96)	0.33 (0.05–2.24)
GI	5 (5.1%)	2 (4.2%)		
GU	0 (0.0%)	0 (0.0%)		
Haematological	2 (2.0%)	1 (2.1%)		
Fatigue/pain	1 (1.0%)	0 (0.0%)		
Dermatological/neurological	1 (1.0%)	0 (0.0%)		
Vaginal	0 (0.0%)	0 (0.0%)		
Late toxicities				
Any	13 (13.3%)	2 (4.4%)	0.30 (0.06–1.38)	0.37 (0.07–1.81)
GI	9 (9.2%)	2 (4.4%)		
GU	3 (3.0%)	0 (0.0%)		
Haematological	2 (2.0%)	0 (0.0%)		
Fatigue/pain	1 (1.0%)	0 (0.0%)		
Dermatological/neurological	0 (0.0%)	0 (0.0%)		
Vaginal	1 (1.0%)	0 (0.0%)		

2D-BT = 2D brachytherapy, 3D-IGABT = 3D Image-guided adaptive brachytherapy. ^1^ Adjusted for: age (continuous), histology (categorical), stage (1/2/3/4, continuous), lymph node involvement (binary), EBRT dose (categorical), and brachytherapy dose (categorical). Uni- and multivariable logistic regression models were used to calculate odds ratios and their respective 95% confidence intervals. An odds ratio above 1 means more grade ≥ 3 toxicities in the 3D-IGABT group, and below 1 less grade ≥ 3 toxicities.

**Table 4:** Baseline variables associated with disease control and survival outcomes.

	Locoregional Control	Distant Control	Failure-Free Survival	Cancer-Specific Survival	Overall Survival
Univariable analysis					
Choice of brachytherapy (3D-IGABT; ref: 2D-BT)	0.72 (0.20–2.62)	1.57 (0.79–3.13)	1.23 (0.65–2.32)	1.07 (0.44–2.58)	0.96 (0.42–2.16)
Age at diagnosis (per 5-year increment)	**0.74** **(0.57–0.95)**	1.00 (0.88–1.13)	0.96 (0.86–1.08)	1.03 (0.89–1.19)	1.10 (0.97–1.25)
Histology (non-SCC; ref: SCC)	1.35 (0.30–6.06)	2.02 (0.89–4.63)	1.78 (0.83–3.83)	1.04 (0.31–3.48)	1.47 (0.56–3.83)
Clinical stage (per stage increment)	1.34 (0.73–2.44)	**1.98** **(1.40–2.81)**	**1.97** **(1.43–2.70)**	**2.48** **(1.66–3.72)**	**2.16** **(1.50–3.09)**
Location of positive lymph nodes (ref: negative lymph nodes)					
− Pelvic	0.95 (0.26–3.55)	**4.18** **(1.22–14.35)**	2.28 (0.96–5.38)	**7.97** **(1.03–61.75)**	3.13 (0.89–10.99)
− Para-aortic/retroperitoneal	1.32 (0.30–5.91)	**8.96** **(2.55–31.50)**	**3.80** **(1.51–9.55)**	**19.42** **(2.50–150.6)**	**7.56** **(2.15–26.60)**
EBRT dose (>50.4 Gy; ref: ≤50.4 Gy)	0	1.82 (0.71–4.67)	1.39 (0.55–3.50)	0.44 (0.06–3.28)	0.35 (0.05–2.58)
BT dose (<24 Gy; ref ≥24 Gy)	0	0.78 (0.24–2.55)	0.62 (0.19–2.01)	1.13 (0.34–3.75)	1.18 (0.42–3.37)
Concurrent chemotherapy					
− Cisplatin	1.23 (0.16–9.40)	0.59 (0.23–1.51)	0.58 (0.25–1.37)	0.41 (0.15–1.10)	**0.36** **(0.15–0.83)**
− Gemcitabine	2.97 (0.39–22.80)	2.73 (0.65–11.49)	**3.41** **(1.05–11.10)**	3.58 (0.84–15.24)	2.73 (0.65–11.49)
Multivariable analysis					
Choice of brachytherapy (3D-IGABT; ref: 2D-BT)	0.81 (0.21–3.13)	1.94 (0.85–4.40)	1.44 (0.70–2.98)	1.02 (0.38–2.75)	0.82 (0.33–2.02)
Age at diagnosis (per 5-year increment)	**0.66** **(0.46–0.94)**	0.90 (0.74–1.09)	0.87 (0.73–1.04)	1.03 (0.82–1.29)	1.13 (0.94–1.36)
Histology (non-SCC; ref: SCC)	1.26 (0.25–6.28)	1.96 (0.67–5.73)	1.63 (0.63–4.24)	1.45 (0.39–5.47)	1.55 (0.50–4.86)
Clinical stage (per stage increment)	1.82 (0.91–3.65)	**1.91** **(1.25–2.89)**	**1.90** **(1.31–2.75)**	**1.98** **(1.21–3.26)**	**1.64** **(1.06–2.53)**
Location of positive lymph nodes (ref: negative lymph nodes)					
− Pelvic	0.52 (0.13–2.12)	**3.93** **(1.07–14.45)**	1.93 (0.77–4.87)	**9.21** **(1.12–75.92)**	**4.36** **(1.15–16.61)**
− Para-aortic/retroperitoneal	0.86 (0.18–4.26)	**7.09** **(1.94–25.84)**	**2.68** **(1.01–7.10)**	**11.66** **(1.39–97.88)**	**5.40** **(1.39–20.99)**
EBRT dose (>50.4 Gy; ref: ≤50.4 Gy)	N/A	2.44 (0.83–7.15)	1.77 (0.63–4.94)	0.49 (0.06–3.88)	0.33 (0.04–2.52)
BT dose (<24 Gy; ref ≥24 Gy)	N/A	1.53 (0.40–5.86)	1.36 (0.37–5.02)	1.46 (0.37–5.76)	1.76 (0.53–5.59)
Concurrent chemotherapy					
− Cisplatin	0.32 (0.02–4.36)	0.34 (0.10–1.23)	**0.20** **(0.06–0.66)**	**0.15** **(0.04–0.60)**	**0.19** **(0.06–0.62)**
− Gemcitabine	3.43 (0.22–53.00)	1.56 (0.30–8.02)	2.22 (0.58–8.42)	2.48 (0.33–18.66)	2.90 (0.44–19.07)

2D-BT, two-dimensional brachytherapy; 3D-IGABT, three-dimensional image-guided adaptive brachytherapy; EBRT, external beam radiotherapy; Gy, Gray; ref, reference; SCC, squamous cell carcinoma. Values are hazard ratios with 95% confidence intervals, as calculated using uni- and multivariable Cox proportional-hazards models. In the multivariable analyses, we adjusted for choice of brachytherapy, age, histology, clinical stage, location of positive lymph nodes, and EBRT/BT dose. Significant values (*p* < 0.05) are marked in bold.

## Data Availability

The data from this study are available from the corresponding author, J.A., upon request.

## Supplementary Materials

The following supporting information can be downloaded at: https://www.mdpi.com/article/10.3390/curroncol30050375/s1, Figure S1: Higher clinical stage significantly predicts for worse overall survival; Table S1: Life tables for local and distant recurrences, progression-free survival and overall survival.
