# Supplementary material for: Safety and Efficacy of 2D Brachytherapy vs. 3D Image-Guided Adaptive Brachytherapy for Locally Advanced Cervical Cancer—A Single Institution Retrospective Study
Source: Curr Oncol. 2023 May 13;30(5):4966–78. doi: 10.3390/curroncol30050375 (PMC10217529; doi:10.3390/curroncol30050375)
Supplement: Supplementary file 1 [file curroncol-30-00375-s001.zip › curroncol-2332109-supplementary.pdf]

Supplementary Table S1: Life tables for local and distant recurrences, progression-free survival and overall survival.

	Local recurrence		Distant recurrence	
	**ICRU**	**3D**	**ICRU**	**3D**
**1 year**	5.7% (2.4-13.0%)	4.8% (1.2-17.7%)	13.2% (7.7-22.1%)	18.8% (9.9-34.1%)
**2 years**	11.0% (5.9-20.2%)	8.1% (2.6-23.3%)	18.2% (11.5-28.0%)	31.6% (19.1-49.3%)
**3 years**	11.0% (5.9-20.2%)	8.0% (2.6-23.3%)	23.7% (15.9-34.4%)	36.6% (22.4-56.0%)
**4 years**	12.7% (7.0-22.6%)	8.0% (2.6-23.3%)	28.2% (19.6-39.6%)	36.6% (22.4-56.0%)
**5 years**	12.7% (7.0-22.6%)	8.0% (2.6-23.3%)	28.2% (19.6-39.6%)	36.6% (22.4-56.0%)
**6 years**	12.7% (7.0-22.6%)	8.0% (2.6-23.3%)	28.2% (19.6-39.6%)	36.6% (22.4-56.0%)

	Locoregional control		Distant control	
	**ICRU**	**3D**	**ICRU**	**3D**
**3 years**	89.0% (79.8-94.1%)	92.0% (76.7-97.4%)	76.3% (65.6-84.1%)	63.4% (44.0-77.6%)
**5 years**	87.3% (77.4-93.0%)	92.0% (76.7-97.4%)	71.8% (60.4-80.4%)	63.4% (44.0-77.6%)

	FFS		OS	
	**ICRU**	**3D**	**ICRU**	**3D**
**1 year**	82.6% (73.2-89.0%)	79.1% (63.6-88.5%)	90.3% (82.2-94.8%)	90.8% (77.3-96.5%)
**2 years**	73.0% (62.4-81.0%)	66.7% (49.2-79.3%)	83.2% (73.7-89.6%)	87.8% (73.0-94.8%)
**3 years**	69.1% (58.2-77.7%)	61.7% (42.8-76.0%)	76.7% (66.2-84.4%)	73.6% (52.1-86.6%)
**4 years**	63.7% (52.4-73.0%)	61.7% (42.8-76.0%)	72.4% (61.3-80.8%)	73.6% (52.1-86.6%)
**5 years**	63.7% (52.4-73.0%)	61.7% (42.8-76.0%)	70.8% (59.4-79.5%)	73.6% (52.1-86.6%)
**6 years**	63.7% (52.4-73.0%)	61.7% (42.8-76.0%)	70.8% (59.4-79.5%)	73.6% (52.1-86.6%)

Supplementary Figure S1: Higher clinical stage significantly predicts for worse overall survival.

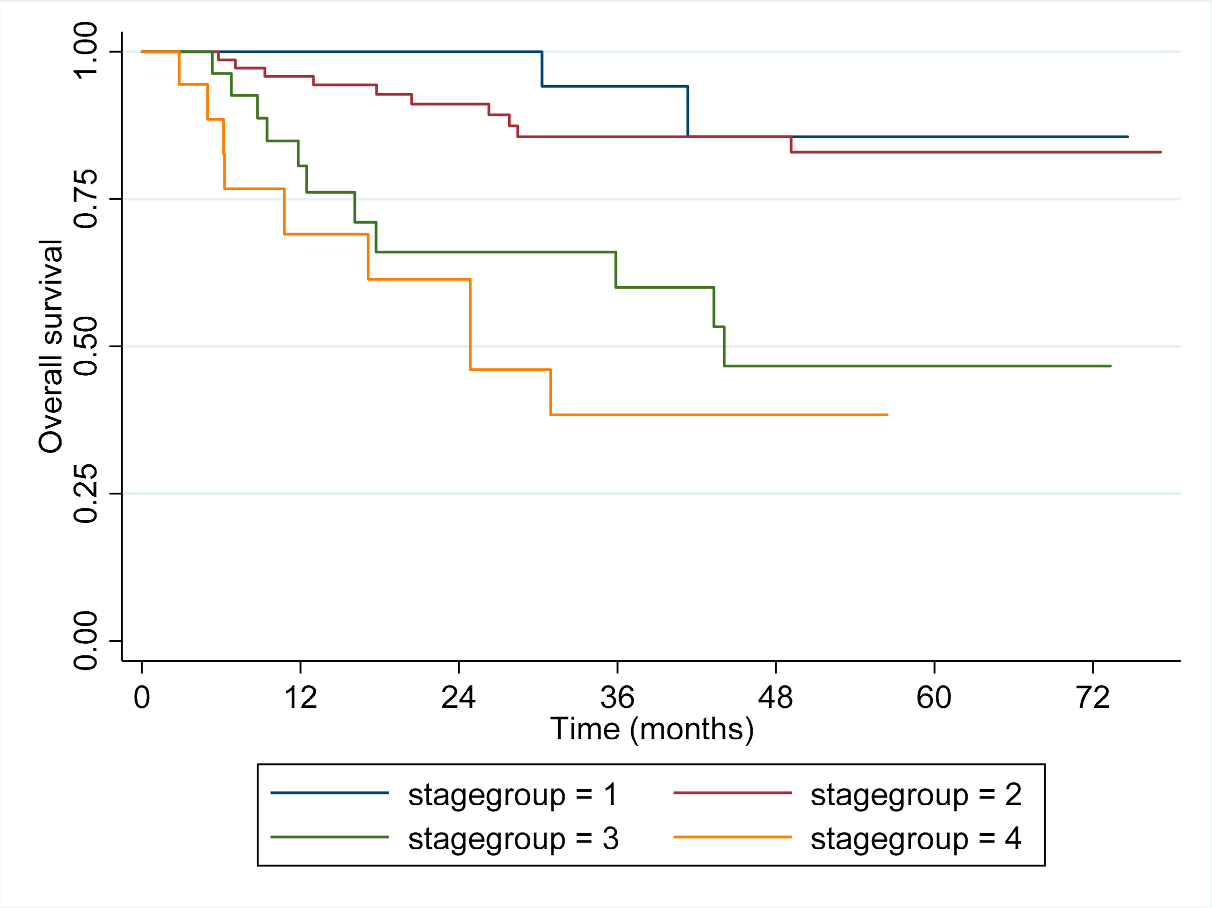

Death events were recorded per stage group and analyzed by multivariable Cox regression adjusted for choice of BT technique, age, histology, location of positive lymph nodes, and EBRT/BT dose. Hazard ratio = 1.64 (95% CI 1.06-2.53)
